# COVID 19 vaccine in the pediatric age: the recommendation of the Italian Pediatric Society

**DOI:** 10.1186/s13052-022-01244-3

**Published:** 2022-03-24

**Authors:** Annamaria Staiano, Rino Agostiniani, Elena Bozzola, Rocco Russo, Giovanni Corsello

**Affiliations:** The Italian Pediatric Society, Rome, Italy

**Keywords:** Children, Covid-19 vaccine, Hesitancy

## Abstract

Vaccine is an important and effective tool to protect from preventable infectious diseases. Neverthless, in the COVID-19 pandemic era, scientific and accurate information are required to responde to false and misleading information on efficacy and safety of immunization in the pediatric age.

## Background

Vaccine has been widely considered as an important and effective tool to protect from preventable infectious diseases. Scientific and accurate information are required to get people informed and to contribute avoiding common myths and rumors based on misinformation [1]. The European Pediatric Association, as well as the American Academy of Pediatrics, strongly recommends COVID-19 immunization in 5–11 years old children to reduce the burden of COVID-19, prevent severe COVID-19 disease and avoid neuropsichological consequences of quarantine and isolation among the youngest [2].

## Main text

The Italian Pediatric Society promote the vaccination in children over 5 years, fighting against false information and unscientific statements and promoting a detailed and accurate answer to families’doubts. The most relevant information to share with the community are reported in the following bulletin.

1. As for COVID-19 vaccine, the most frequent concern from families is on the safeness for children aged 5–11 years [3]. While COVID-19 vaccines were developed rapidly, all steps were taken to make sure they are safe and effectivive for children. During the development of COVID-19 vaccines, phases overlapped to speed up the process, but all phases were completed as required for a clinical trial. COVID-19 vaccine for children ages 5 through 11 years were developed and tested in the same way as adult COVID-19 vaccines. In clinical trial, vaccine side effects were examined and appeared to be mild, similar to those seen with other vaccines recommended in infancy [4]. Data produced by the industry has been reviewed and analysed by Food and Drug Administration in the United States of America, by European Medicines Agency in Europe and Italian Medicines Agency in our Country. So, actually, the vaccine can be considered safe and effective to the target population who will receive it, namely children aged 5–11 years. To enforce this consideration, over 5 milions of children in the world already got one and in some cases two shots, without safety concerns. Over 7 millions of COVID 19 vaccine shot, just 8 myocarditis have been demonstrated correlated to the immunization. All the children had mild symptoms and promptly recovered. Unlike the mild side effects that some may experience after vaccination, children who got infected with COVID-19 are at risk of getting seriously ill and being hospitalized. From the beginning of COVID-19 pandemic to the 21^st^ December 2021, in Italy children ages 5 through 11 years have experienced 954.657 COVID-19 cases and 35 deaths from COVID-19. From mid October 2021, a severe increase of cases in 6–11 years old children has been documented, mainly in the latest two weeks. In details, in the period 6–19 December 2021, 59.605 cases have been notifed in children 5–11 years aged, of whom 215 required hospital admission and 4 intensive unit care [5].
2. Getting children 5 years and older vaccinated can help protect them from getting COVID-19, as well as keep them in school and group activities.
3. Moreover, as suggested by the American Academy of Pediatrics, COVID-19 vaccination is the advisable way to protect children not only from the infection but also from disease’s short and long term consequences, such as long Covid-19 and multisystem inflammatory syndrome [6]. In fact, even children who develop mild symptoms may be affected by sequelae weeks or months after recovering. Getting COVID -19 even if with mild symptoms is not advisable as it does neither guarantee a stronger immunity nor allow a long protection against the virus. It has been demonstrated that people who got an infection maybe re-infected later in life. In children who have had prior symptomatic or asymptomatic SARS-CoV-2 infection, the administration of a single dose of SARS-CoV-2/COVID-19 vaccine may be considered, preferably within 6 months from the infection and no later than 12 months after its resolution. A 2 dose schedule is suggested if the infection happened more than one year before the infection.
4. Finally, children vaccination contribute to stop the spread of COVID-19 in the community and may help protecting those unvaccinable for age or for underlying medical conditions.
5. As for safety concern, one of the mayor doubt is on pubertal development and fertility. There is neither evidence nor plausible scientific base to state that any vaccines, including COVID-19 vaccines, may interfere with puberty and female or male fertility. To date, there is no evidence that vaccine ingredients or antibodies developed following COVID-19 vaccination may interfere with pregnancy. On the contrary, evidence suggested that females who received COVID-19 vaccine met no problems in becoming pregnant [4, 7].

## Conclusions

Vaccine hesitancy may interfere with universal children immunization. Answering to families’ doubts about vaccine efficacy, safety and real need may represent a winning strategy to promote children immunization.

## Data Availability

Data supporting the considerations reported in the text can be found at Dr. Bozzola’s repository.
